# A dermatopathic Juvenile Dermatomyositis; An Unexpected Case in Childhood

**Published:** 2020

**Authors:** Mehrnoush HASSAS YEGANEH, Pooria AHMADI, Yalda NILIPOUR, Mohadese sadat MOUSAVI KHORSHIDI, Reza SINAEI, Mohammad Reza FATHI, Reza SHIARI

**Affiliations:** 1 Department of Pediatrics, Division of Pediatric Rheumatology, Shahid Beheshti University of Medical Sciences, Tehran- Iran; 2 Medical School, Shahid Beheshti University of Medical Sciences , Tehran, Iran.; 3 Padiatric Pathology Research center, research institute for children’s health, Shahid Beheshti University of Medical Sciences, Tehran, Iran; 4 Department of Pediatric, Shahid Beheshti University of Medical Sciences, Tehran, Iran; 5 Pediatric Rheumatology, Kerman University of Medical Sciences, Kerman, Iran; 6 Pediatric Rheumatology, Ahwaz Jundishapur University of Medical Sciences, Ahvaz, Iran

**Keywords:** Dermatopathic, Juvenile Dermatomyositis, TreatmentIntroduction

## Abstract

Juvenile dermatomyositis (JDM) is a rare idiopathic inflammatory disease, which usually presents with skin rashes along with muscle weakness. We report a case of JDM in a 10- year-old girl with no skin manifestations presenting with progressive muscle weakness and fatigue. Further laboratory investigations, along with a muscle biopsy, confirmed the diagnosis of adermatopathic JDM. The patient was treated with intravenous immunoglobulin, corticosteroids, methotrexate, hydroxychloroquine, pamidronate, and rituximab.

Following treatment, patients’ symptoms subsided, and she gained normal muscular strength over a year.

## Introduction

Juvenile dermatomyositis (JDM) is a multisystem disease of uncertain origin that is defined by chronic inflammation of striated muscle and skin in children less than 16 years of age(1). Perifascicular atrophy is the typical muscular and vascular histologic outcome of JDM. Amyopathic dermatomyositis is an uncommon variant of JDM, characterized by the hallmark cutaneous features of dermatomyositis for at least 6 months without clinical or laboratory evidence of muscle disease (2). Cases of dermatomyositis with absent skin findings have been reported and are referred to as adermatopathic dermatomyositis (2). Herein, we present the findings of a patient with adermatopathic JDM.

## Case Presentation 

A 10-year-old girl presented with had upper limb pain from shoulder to wrist and lower limb pain from hip to knee that has lasted three months. She had progressive proximal limb weakness to the extent that she could barely walk five steps and was unable to comb her hair. She also was able to feed herself, but she gets tired very quickly and had a history of remittent fever. The patient had no complaints of constipation, diarrhea, or any abdominal pain.On initial assessment, she was afebrile, and the examinations showed diffused tender, warm muscles with proximal limb weakness, and medical research council (MRC) scale for muscle strength grade 2 for upper and lower limbs. Her shoulder had limited passive range of motion due to pain, and her knees and hip joints were tender. Deep tendon reflexes were present and normal.The Gottron’s papules and heliotrope rashes were absent, and no skin involvement was found. Fingers and nails were found to be normal.Further investigations yielded the following results: Creatine Phosphokinase (CPK): 3047 U/L, Alanine Aminotransferase (ALT): 81 U/L, Aspartate Aminotransferase (AST): 173 U/L, Lactate Dehydrogenase (LDH): 1271 U/L, Erythrocyte Sedimentation Rate (ESR): 16 mm/hr, Anti-nucleotide Antibody (ANA): 0.4, and 25-hydroxycholecalciferol (VitD): 8 ng/mL. Urine analysis showed leukocyturia, urine culture showed 30000 30,000 colonies of two types, and the results were negative for C-Reactive Protein (CRP), Rheumatoid Factor (RF), 2-Mercapthoethanol (2ME), and wright. Electromyography and Nerve Conduction Velocity (EMG/NCV) findings were consistent with a myogenic process; therefore, a muscle biopsy was performed. Deltoid muscle biopsy was performed, and the specimen was sent to be frozen in Isopentane cooled in liquid nitrogen. A panel of histology and histochemistry studies revealed a perifascicular atrophy pattern with the presence of atrophic basophilic degenerative/regenerative fibers at the periphery of the fascicles containing internalized nuclei (Figure 1a). Perimysial perivascular infiltration of lymphocytes was also noted (Figure 1b). Based on these pathognomonic findings associated with the patient’s phenotype, the diagnosis of JDM was made.

The patient was hospitalized to receive six courses of treatment. Intravenous Immunoglobulin (IVIG) therapy was initiated (2 gr/Kg per session) and was monthly repeated for 6 months. Methylprednisolone (30 mg/Kg) was also administered intravenously for 3 consequent days. Treatment followed by oral Prednisolone, Methotrexate, Hydroxychloroquine, Folic Acid, Calcium, and Vitamin D_3_ Supplements as maintenance therapy. The treatment did not change her distal upper and lower limb weakness; however, proximal upper limb weakness was resolved. After six months of treatment using IVIG and steroid, the lower limb weakness worsened (MRC grade 4), and areflexia of patellar tendon was observed. No signs of lower limb polyneuropathies were found on further neurological assessment.

Our patient showed signs of Cushing’s syndrome (purple striae, moon face, osteopenia, and fragile skin) two months after initiating treatment with corticosteroids, and she complained of lumbosacral pain.

Therefore, treatment with Rituximab (500 mg/m^2^) was started followed by other complementary doses. Her condition was markedly improved, and she was discharged with the aforementioned routine prescriptions to be followed up.

**Figure1a:**
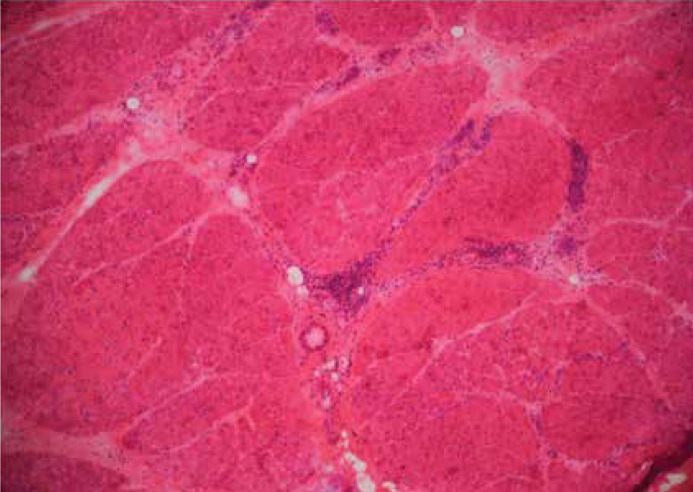
Multiple foci of perivascular perimysial chronic inflammation (H&E stain *40)

**Figure1b:**
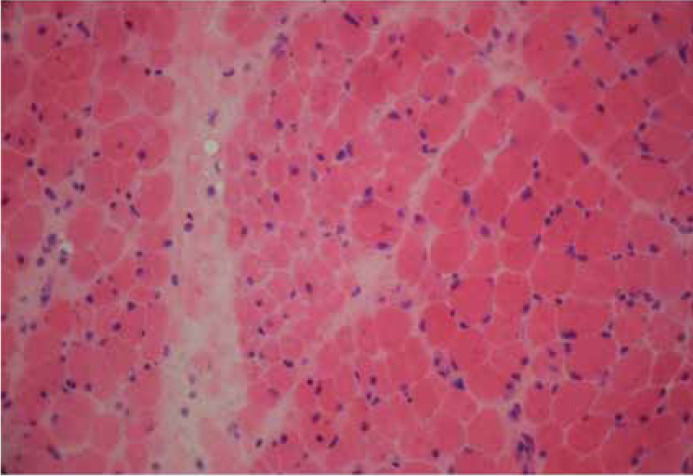
Typical perifascicular atrophy pattern (H&E stain*400)

## Discussion

Dermatomyositis is a chronic inflammation of unknown origin involving the musculoskeletal system. Chronic Idiopathic Inflammatory Myopathies (IIMs) in children are a heterogeneous group of disorders. Although many affected children have muscle and skin abnormalities similar to JDM, it can be diagnosed by the five criteria developed by Bohan and Peter (4, 3). Diagnosis of JDM often requires the presence of the pathognomonic rashes (Heliotrope rash or Gottron papules over the extensor surfaces) and two other criteria (4, 3); however, due to the cases of dermatomyositis in adults without skin involvement (2), a different subtype, adermatopathic dermatomyositis, has been postulated. It is defined by the absence of skin rashes along with myositis plus either perifascicular atrophy at muscle biopsy or pathognomonic rashes emerging in the follow-up or calcinosis in dermatomyositis (5). To our knowledge, there is no reported case of adermatopathic dermatomyositis in childhood. Rituximab has been shown effective against severe/refractory cases of JDM; therefore, it was used to treat the patient and showed favorable outcomes.

**Inconclusion,** In our experience, treatment with intravenous immunoglobulin injections, methotrexate, hydroxychloroquine, and corticosteroids showed no satisfactory improvement inpatient with adermatopathic dermatomyositis. However, using the biologic Rituximab was effective to control and treat the disorder.
